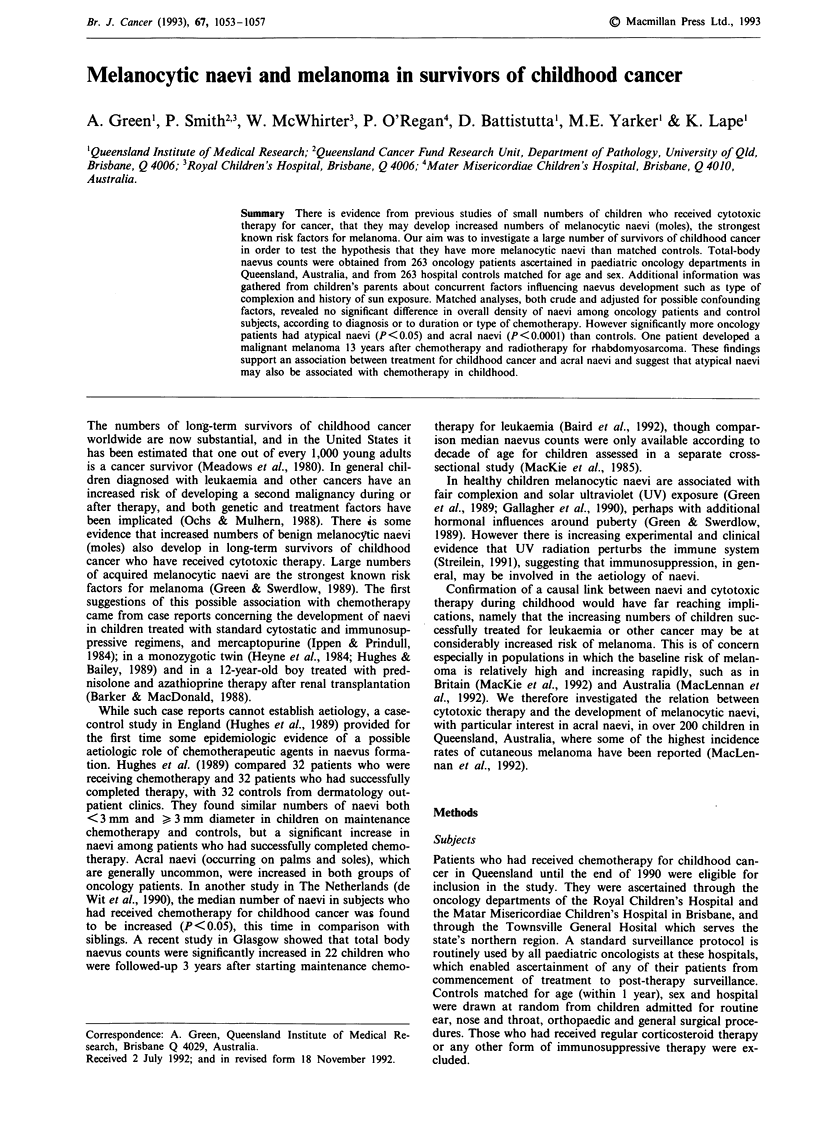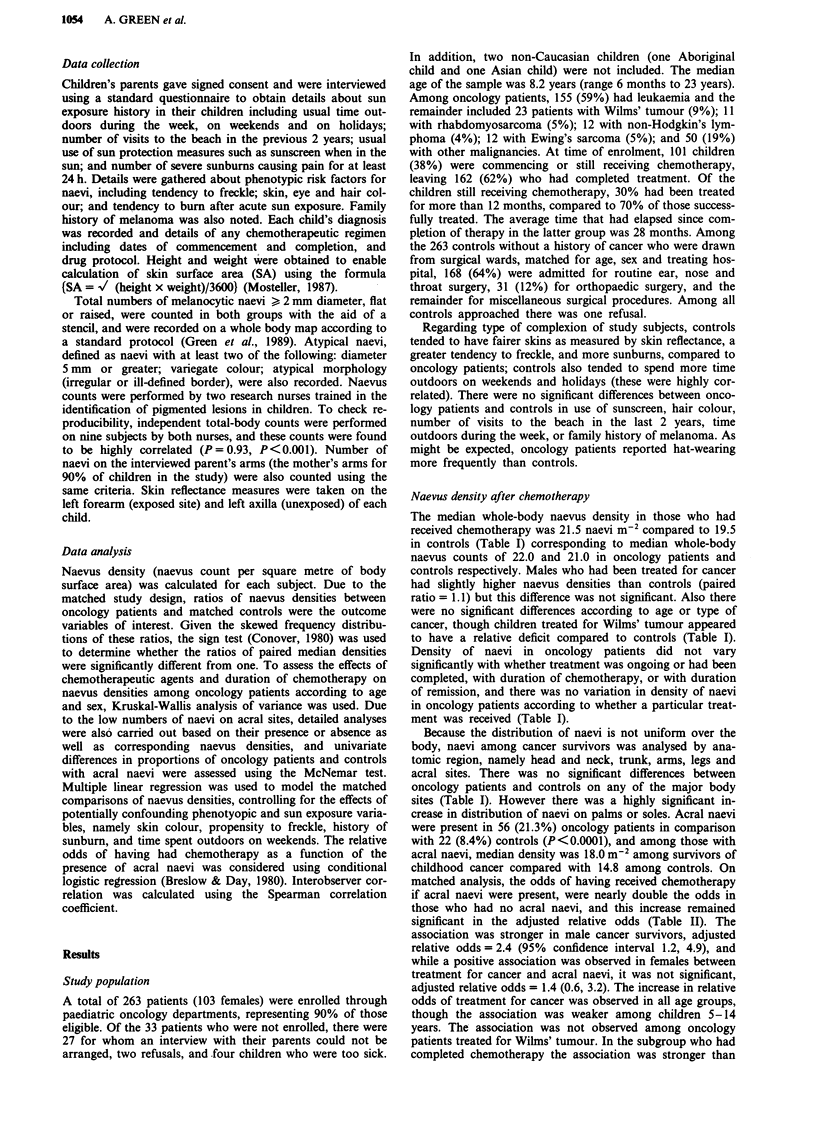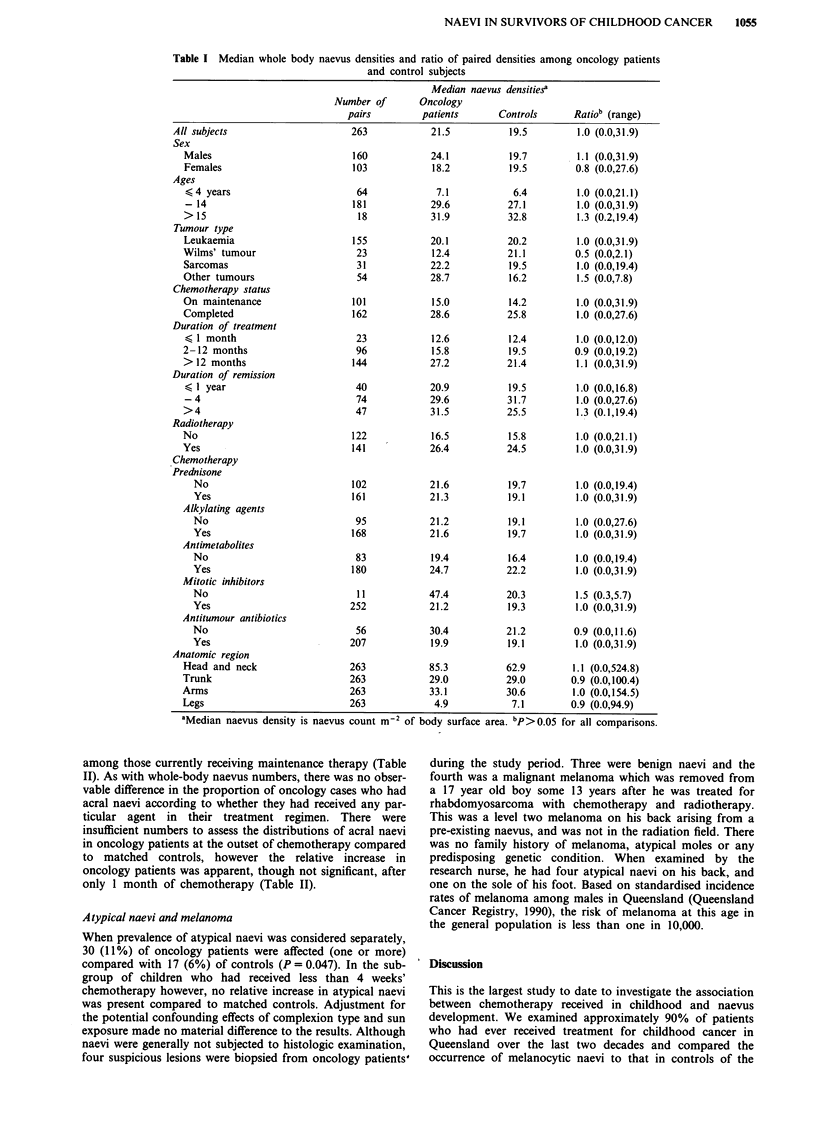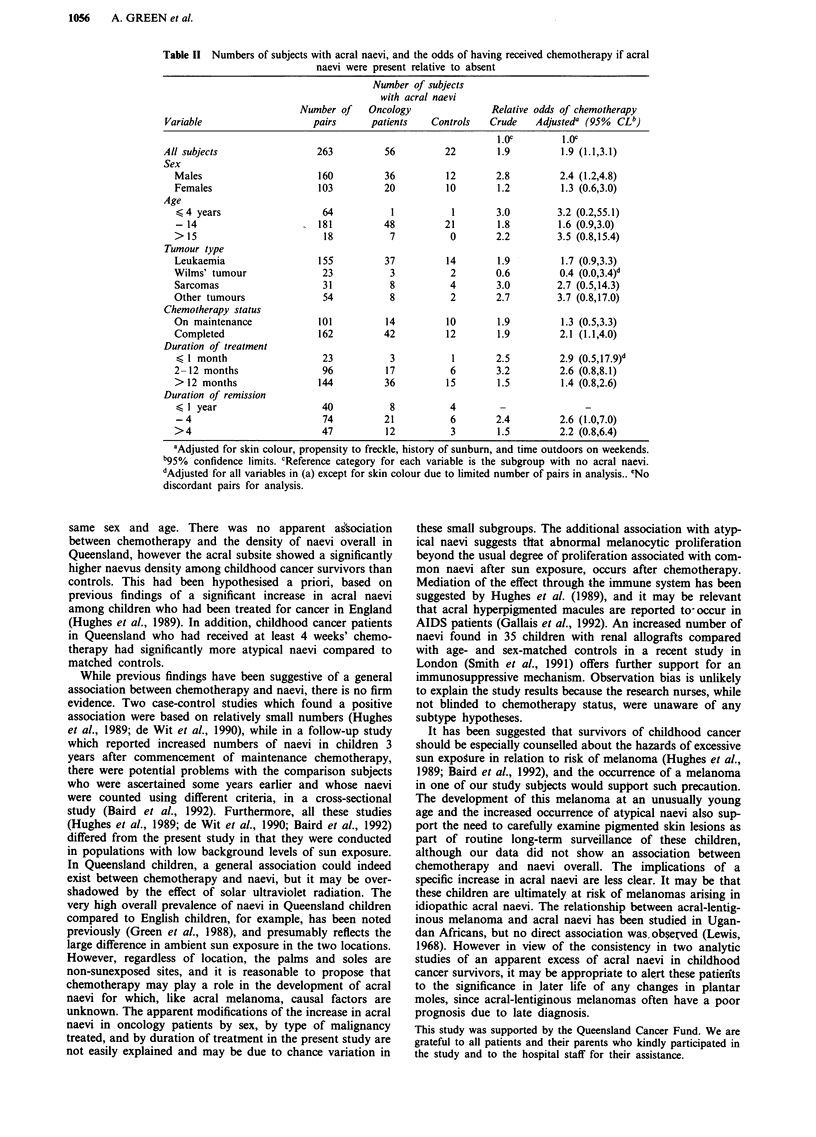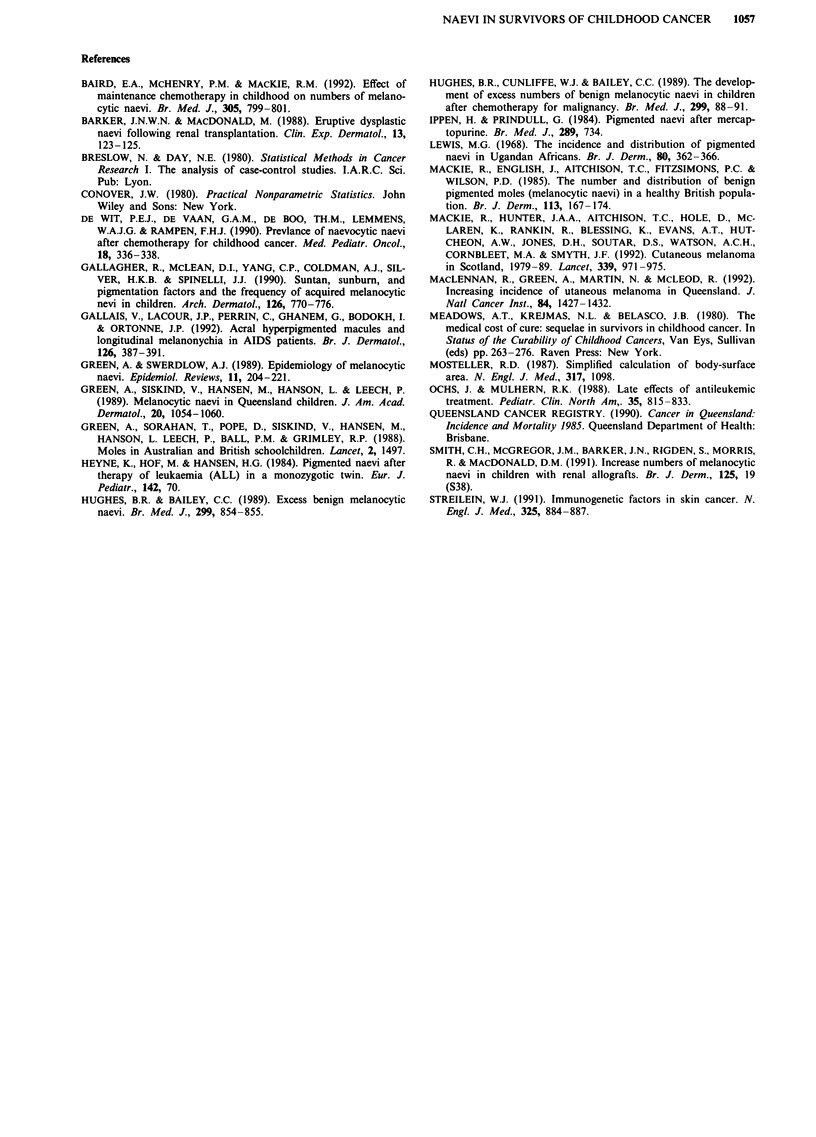# Melanocytic naevi and melanoma in survivors of childhood cancer.

**DOI:** 10.1038/bjc.1993.193

**Published:** 1993-05

**Authors:** A. Green, P. Smith, W. McWhirter, P. O'Regan, D. Battistutta, M. E. Yarker, K. Lape

**Affiliations:** Queensland Institute of Medical Research, University of Qld, Brisbane, Australia.

## Abstract

There is evidence from previous studies of small numbers of children who received cytotoxic therapy for cancer, that they may develop increased numbers of melanocytic naevi (moles), the strongest known risk factors for melanoma. Our aim was to investigate a large number of survivors of childhood cancer in order to test the hypothesis that they have more melanocytic naevi than matched controls. Total-body naevus counts were obtained from 263 oncology patients ascertained in paediatric oncology departments in Queensland, Australia, and from 263 hospital controls matched for age and sex. Additional information was gathered from children's parents about concurrent factors influencing naevus development such as type of complexion and history of sun exposure. Matched analyses, both crude and adjusted for possible confounding factors, revealed no significant difference in overall density of naevi among oncology patients and control subjects, according to diagnosis or to duration or type of chemotherapy. However significantly more oncology patients had atypical naevi (P < 0.05) and acral naevi (P < 0.0001) than controls. One patient developed a malignant melanoma 13 years after chemotherapy and radiotherapy for rhabdomyosarcoma. These findings support an association between treatment for childhood cancer and acral naevi and suggest that atypical naevi may also be associated with chemotherapy in childhood.


Br. J. Cancer (1993), 67, 1053-1057
© Macmillan Press Ltd., 1993

### Melanocytic naevi and melanoma in survivors of childhood cancer

A. Green[1], P. Smith[2,3], W. McWhirter[3], P. O'Regan[4], D. Battistutta[1], M.E. Yarker[1] & K. Lape[1]

[1]*Queensland Institute of Medical Research;* [2]*Queensland Cancer Fund Research Unit, Department of Pathology, University of Qld, Brisbane, Q 4006;* [3]*Royal Children's Hospital, Brisbane, Q 4006;* [4]*Mater Misericordiae Children's Hospital, Brisbane, Q 4010, Australia.*

**Summary** There is evidence from previous studies of small numbers of children who received cytotoxic therapy for cancer, that they may develop increased numbers of melanocytic naevi (moles), the strongest known risk factors for melanoma. Our aim was to investigate a large number of survivors of childhood cancer in order to test the hypothesis that they have more melanocytic naevi than matched controls. Total-body naevus counts were obtained from 263 oncology patients ascertained in paediatric oncology departments in Queensland, Australia, and from 263 hospital controls matched for age and sex. Additional information was gathered from children's parents about concurrent factors influencing naevus development such as type of complexion and history of sun exposure. Matched analyses, both crude and adjusted for possible confounding factors, revealed no significant difference in overall density of naevi among oncology patients and control subjects, according to diagnosis or to duration or type of chemotherapy. However significantly more oncology patients had atypical naevi ($P<0.05$) and acral naevi ($P<0.0001$) than controls. One patient developed a malignant melanoma 13 years after chemotherapy and radiotherapy for rhabdomyosarcoma. These findings support an association between treatment for childhood cancer and acral naevi and suggest that atypical naevi may also be associated with chemotherapy in childhood.

The numbers of long-term survivors of childhood cancer worldwide are now substantial, and in the United States it has been estimated that one out of every 1,000 young adults is a cancer survivor (Meadows *et al.*, 1980). In general children diagnosed with leukaemia and other cancers have an increased risk of developing a second malignancy during or after therapy, and both genetic and treatment factors have been implicated (Ochs & Mulhern, 1988). There is some evidence that increased numbers of benign melanocytic naevi (moles) also develop in long-term survivors of childhood cancer who have received cytotoxic therapy. Large numbers of acquired melanocytic naevi are the strongest known risk factors for melanoma (Green & Swerdlow, 1989). The first suggestions of this possible association with chemotherapy came from case reports concerning the development of naevi in children treated with standard cytostatic and immunosuppressive regimens, and mercaptopurine (Ippen & Prindull, 1984); in a monozygotic twin (Heyne *et al.*, 1984; Hughes & Bailey, 1989) and in a 12-year-old boy treated with prednisolone and azathioprine therapy after renal transplantation (Barker & MacDonald, 1988).

While such case reports cannot establish aetiology, a case-control study in England (Hughes *et al.*, 1989) provided for the first time some epidemiologic evidence of a possible aetiologic role of chemotherapeutic agents in naevus formation. Hughes *et al.* (1989) compared 32 patients who were receiving chemotherapy and 32 patients who had successfully completed therapy, with 32 controls from dermatology outpatient clinics. They found similar numbers of naevi both $<3$ mm and $\geqslant 3$ mm diameter in children on maintenance chemotherapy and controls, but a significant increase in naevi among patients who had successfully completed chemotherapy. Acral naevi (occurring on palms and soles), which are generally uncommon, were increased in both groups of oncology patients. In another study in The Netherlands (de Wit *et al.*, 1990), the median number of naevi in subjects who had received chemotherapy for childhood cancer was found to be increased ($P<0.05$), this time in comparison with siblings. A recent study in Glasgow showed that total body naevus counts were significantly increased in 22 children who were followed-up 3 years after starting maintenance chemotherapy for leukaemia (Baird *et al.*, 1992), though comparison median naevus counts were only available according to decade of age for children assessed in a separate cross-sectional study (MacKie *et al.*, 1985).

In healthy children melanocytic naevi are associated with fair complexion and solar ultraviolet (UV) exposure (Green *et al.*, 1989; Gallagher *et al.*, 1990), perhaps with additional hormonal influences around puberty (Green & Swerdlow, 1989). However there is increasing experimental and clinical evidence that UV radiation perturbs the immune system (Streilein, 1991), suggesting that immunosuppression, in general, may be involved in the aetiology of naevi.

Confirmation of a causal link between naevi and cytotoxic therapy during childhood would have far reaching implications, namely that the increasing numbers of children successfully treated for leukaemia or other cancer may be at considerably increased risk of melanoma. This is of concern especially in populations in which the baseline risk of melanoma is relatively high and increasing rapidly, such as in Britain (MacKie *et al.*, 1992) and Australia (MacLennan *et al.*, 1992). We therefore investigated the relation between cytotoxic therapy and the development of melanocytic naevi, with particular interest in acral naevi, in over 200 children in Queensland, Australia, where some of the highest incidence rates of cutaneous melanoma have been reported (MacLennan *et al.*, 1992).

### Methods

### *Subjects*

Patients who had received chemotherapy for childhood cancer in Queensland until the end of 1990 were eligible for inclusion in the study. They were ascertained through the oncology departments of the Royal Children's Hospital and the Matar Misericordiae Children's Hospital in Brisbane, and through the Townsville General Hosital which serves the state's northern region. A standard surveillance protocol is routinely used by all paediatric oncologists at these hospitals, which enabled ascertainment of any of their patients from commencement of treatment to post-therapy surveillance. Controls matched for age (within 1 year), sex and hospital were drawn at random from children admitted for routine ear, nose and throat, orthopaedic and general surgical procedures. Those who had received regular corticosteroid therapy or any other form of immunosuppressive therapy were excluded.

Correspondence: A. Green, Queensland Institute of Medical Research, Brisbane Q 4029, Australia.
Received 2 July 1992; and in revised form 18 November 1992.

*Data collection*

Children's parents gave signed consent and were interviewed using a standard questionnaire to obtain details about sun exposure history in their children including usual time outdoors during the week, on weekends and on holidays; number of visits to the beach in the previous 2 years; usual use of sun protection measures such as sunscreen when in the sun; and number of severe sunburns causing pain for at least 24 h. Details were gathered about phenotypic risk factors for naevi, including tendency to freckle; skin, eye and hair colour; and tendency to burn after acute sun exposure. Family history of melanoma was also noted. Each child's diagnosis was recorded and details of any chemotherapeutic regimen including dates of commencement and completion, and drug protocol. Height and weight were obtained to enable calculation of skin surface area (SA) using the formula $\{SA = \sqrt{} \text{ (height} \times \text{weight)}/3600\}$ (Mosteller, 1987).

Total numbers of melanocytic naevi $\geqslant 2$ mm diameter, flat or raised, were counted in both groups with the aid of a stencil, and were recorded on a whole body map according to a standard protocol (Green *et al.*, 1989). Atypical naevi, defined as naevi with at least two of the following: diameter 5 mm or greater; variegate colour; atypical morphology (irregular or ill-defined border), were also recorded. Naevus counts were performed by two research nurses trained in the identification of pigmented lesions in children. To check reproducibility, independent total-body counts were performed on nine subjects by both nurses, and these counts were found to be highly correlated ($P = 0.93$, $P < 0.001$). Number of naevi on the interviewed parent's arms (the mother's arms for 90% of children in the study) were also counted using the same criteria. Skin reflectance measures were taken on the left forearm (exposed site) and left axilla (unexposed) of each child.

*Data analysis*

Naevus density (naevus count per square metre of body surface area) was calculated for each subject. Due to the matched study design, ratios of naevus densities between oncology patients and matched controls were the outcome variables of interest. Given the skewed frequency distributions of these ratios, the sign test (Conover, 1980) was used to determine whether the ratios of paired median densities were significantly different from one. To assess the effects of chemotherapeutic agents and duration of chemotherapy on naevus densities among oncology patients according to age and sex, Kruskal-Wallis analysis of variance was used. Due to the low numbers of naevi on acral sites, detailed analyses were also carried out based on their presence or absence as well as corresponding naevus densities, and univariate differences in proportions of oncology patients and controls with acral naevi were assessed using the McNemar test. Multiple linear regression was used to model the matched comparisons of naevus densities, controlling for the effects of potentially confounding phenotyopic and sun exposure variables, namely skin colour, propensity to freckle, history of sunburn, and time spent outdoors on weekends. The relative odds of having had chemotherapy as a function of the presence of acral naevi was considered using conditional logistic regression (Breslow & Day, 1980). Interobserver correlation was calculated using the Spearman correlation coefficient.

### Results

*Study population*

A total of 263 patients (103 females) were enrolled through paediatric oncology departments, representing 90% of those eligible. Of the 33 patients who were not enrolled, there were 27 for whom an interview with their parents could not be arranged, two refusals, and four children who were too sick. In addition, two non-Caucasian children (one Aboriginal child and one Asian child) were not included. The median age of the sample was 8.2 years (range 6 months to 23 years). Among oncology patients, 155 (59%) had leukaemia and the remainder included 23 patients with Wilms' tumour (9%); 11 with rhabdomyosarcoma (5%); 12 with non-Hodgkin's lymphoma (4%); 12 with Ewing's sarcoma (5%); and 50 (19%) with other malignancies. At time of enrolment, 101 children (38%) were commencing or still receiving chemotherapy, leaving 162 (62%) who had completed treatment. Of the children still receiving chemotherapy, 30% had been treated for more than 12 months, compared to 70% of those successfully treated. The average time that had elapsed since completion of therapy in the latter group was 28 months. Among the 263 controls without a history of cancer who were drawn from surgical wards, matched for age, sex and treating hospital, 168 (64%) were admitted for routine ear, nose and throat surgery, 31 (12%) for orthopaedic surgery, and the remainder for miscellaneous surgical procedures. Among all controls approached there was one refusal.

Regarding type of complexion of study subjects, controls tended to have fairer skins as measured by skin reflectance, a greater tendency to freckle, and more sunburns, compared to oncology patients; controls also tended to spend more time outdoors on weekends and holidays (these were highly correlated). There were no significant differences between oncology patients and controls in use of sunscreen, hair colour, number of visits to the beach in the last 2 years, time outdoors during the week, or family history of melanoma. As might be expected, oncology patients reported hat-wearing more frequently than controls.

*Naevus density after chemotherapy*

The median whole-body naevus density in those who had received chemotherapy was 21.5 naevi $m^{-2}$ compared to 19.5 in controls (Table I) corresponding to median whole-body naevus counts of 22.0 and 21.0 in oncology patients and controls respectively. Males who had been treated for cancer had slightly higher naevus densities than controls (paired ratio = 1.1) but this difference was not significant. Also there were no significant differences according to age or type of cancer, though children treated for Wilms' tumour appeared to have a relative deficit compared to controls (Table I). Density of naevi in oncology patients did not vary significantly with whether treatment was ongoing or had been completed, with duration of chemotherapy, or with duration of remission, and there was no variation in density of naevi in oncology patients according to whether a particular treatment was received (Table I).

Because the distribution of naevi is not uniform over the body, naevi among cancer survivors was analysed by anatomic region, namely head and neck, trunk, arms, legs and acral sites. There was no significant differences between oncology patients and controls on any of the major body sites (Table I). However there was a highly significant increase in distribution of naevi on palms or soles. Acral naevi were present in 56 (21.3%) oncology patients in comparison with 22 (8.4%) controls ($P < 0.0001$), and among those with acral naevi, median density was 18.0 $m^{-2}$ among survivors of childhood cancer compared with 14.8 among controls. On matched analysis, the odds of having received chemotherapy if acral naevi were present, were nearly double the odds in those who had no acral naevi, and this increase remained significant in the adjusted relative odds (Table II). The association was stronger in male cancer survivors, adjusted relative odds = 2.4 (95% confidence interval 1.2, 4.9), and while a positive association was observed in females between treatment for cancer and acral naevi, it was not significant, adjusted relative odds = 1.4 (0.6, 3.2). The increase in relative odds of treatment for cancer was observed in all age groups, though the association was weaker among children 5–14 years. The association was not observed among oncology patients treated for Wilms' tumour. In the subgroup who had completed chemotherapy the association was stronger than

**Table I** Median whole body naevus densities and ratio of paired densities among oncology patients and control subjects

| | Number of pairs | Median naevus densities[a] Oncology patients | Controls | Ratio[b] (range) |
|---|---|---|---|---|
| *All subjects* | 263 | 21.5 | 19.5 | 1.0 (0.0,31.9) |
| *Sex* | | | | |
| Males | 160 | 24.1 | 19.7 | 1.1 (0.0,31.9) |
| Females | 103 | 18.2 | 19.5 | 0.8 (0.0,27.6) |
| *Ages* | | | | |
| ⩽4 years | 64 | 7.1 | 6.4 | 1.0 (0.0,21.1) |
| – 14 | 181 | 29.6 | 27.1 | 1.0 (0.0,31.9) |
| >15 | 18 | 31.9 | 32.8 | 1.3 (0.2,19.4) |
| *Tumour type* | | | | |
| Leukaemia | 155 | 20.1 | 20.2 | 1.0 (0.0,31.9) |
| Wilms' tumour | 23 | 12.4 | 21.1 | 0.5 (0.0,2.1) |
| Sarcomas | 31 | 22.2 | 19.5 | 1.0 (0.0,19.4) |
| Other tumours | 54 | 28.7 | 16.2 | 1.5 (0.0,7.8) |
| *Chemotherapy status* | | | | |
| On maintenance | 101 | 15.0 | 14.2 | 1.0 (0.0,31.9) |
| Completed | 162 | 28.6 | 25.8 | 1.0 (0.0,27.6) |
| *Duration of treatment* | | | | |
| ⩽1 month | 23 | 12.6 | 12.4 | 1.0 (0.0,12.0) |
| 2–12 months | 96 | 15.8 | 19.5 | 0.9 (0.0,19.2) |
| >12 months | 144 | 27.2 | 21.4 | 1.1 (0.0,31.9) |
| *Duration of remission* | | | | |
| ⩽1 year | 40 | 20.9 | 19.5 | 1.0 (0.0,16.8) |
| – 4 | 74 | 29.6 | 31.7 | 1.0 (0.0,27.6) |
| >4 | 47 | 31.5 | 25.5 | 1.3 (0.1,19.4) |
| *Radiotherapy* | | | | |
| No | 122 | 16.5 | 15.8 | 1.0 (0.0,21.1) |
| Yes | 141 | 26.4 | 24.5 | 1.0 (0.0,31.9) |
| *Chemotherapy* | | | | |
| *Prednisone* | | | | |
| No | 102 | 21.6 | 19.7 | 1.0 (0.0,19.4) |
| Yes | 161 | 21.3 | 19.1 | 1.0 (0.0,31.9) |
| *Alkylating agents* | | | | |
| No | 95 | 21.2 | 19.1 | 1.0 (0.0,27.6) |
| Yes | 168 | 21.6 | 19.7 | 1.0 (0.0,31.9) |
| *Antimetabolites* | | | | |
| No | 83 | 19.4 | 16.4 | 1.0 (0.0,19.4) |
| Yes | 180 | 24.7 | 22.2 | 1.0 (0.0,31.9) |
| *Mitotic inhibitors* | | | | |
| No | 11 | 47.4 | 20.3 | 1.5 (0.3,5.7) |
| Yes | 252 | 21.2 | 19.3 | 1.0 (0.0,31.9) |
| *Antitumour antibiotics* | | | | |
| No | 56 | 30.4 | 21.2 | 0.9 (0.0,11.6) |
| Yes | 207 | 19.9 | 19.1 | 1.0 (0.0,31.9) |
| *Anatomic region* | | | | |
| Head and neck | 263 | 85.3 | 62.9 | 1.1 (0.0,524.8) |
| Trunk | 263 | 29.0 | 29.0 | 0.9 (0.0,100.4) |
| Arms | 263 | 33.1 | 30.6 | 1.0 (0.0,154.5) |
| Legs | 263 | 4.9 | 7.1 | 0.9 (0.0,94.9) |

[a]Median naevus density is naevus count $m^{-2}$ of body surface area. [b]$P > 0.05$ for all comparisons.

among those currently receiving maintenance therapy (Table II). As with whole-body naevus numbers, there was no observable difference in the proportion of oncology cases who had acral naevi according to whether they had received any particular agent in their treatment regimen. There were insufficient numbers to assess the distributions of acral naevi in oncology patients at the outset of chemotherapy compared to matched controls, however the relative increase in oncology patients was apparent, though not significant, after only 1 month of chemotherapy (Table II).

### *Atypical naevi and melanoma*

When prevalence of atypical naevi was considered separately, 30 (11%) of oncology patients were affected (one or more) compared with 17 (6%) of controls ($P = 0.047$). In the subgroup of children who had received less than 4 weeks' chemotherapy however, no relative increase in atypical naevi was present compared to matched controls. Adjustment for the potential confounding effects of complexion type and sun exposure made no material difference to the results. Although naevi were generally not subjected to histologic examination, four suspicious lesions were biopsied from oncology patients' during the study period. Three were benign naevi and the fourth was a malignant melanoma which was removed from a 17 year old boy some 13 years after he was treated for rhabdomyosarcoma with chemotherapy and radiotherapy. This was a level two melanoma on his back arising from a pre-existing naevus, and was not in the radiation field. There was no family history of melanoma, atypical moles or any predisposing genetic condition. When examined by the research nurse, he had four atypical naevi on his back, and one on the sole of his foot. Based on standardised incidence rates of melanoma among males in Queensland (Queensland Cancer Registry, 1990), the risk of melanoma at this age in the general population is less than one in 10,000.

### Discussion

This is the largest study to date to investigate the association between chemotherapy received in childhood and naevus development. We examined approximately 90% of patients who had ever received treatment for childhood cancer in Queensland over the last two decades and compared the occurrence of melanocytic naevi to that in controls of the

**Table II** Numbers of subjects with acral naevi, and the odds of having received chemotherapy if acral naevi were present relative to absent

| Variable | Number of pairs | Number of subjects with acral naevi: Oncology patients | Number of subjects with acral naevi: Controls | Relative odds of chemotherapy: Crude | Relative odds of chemotherapy: Adjusted[a] (95% CL[b]) |
|---|---|---|---|---|---|
| | | | | 1.0[c] | 1.0[c] |
| *All subjects* | 263 | 56 | 22 | 1.9 | 1.9 (1.1,3.1) |
| *Sex* | | | | | |
| Males | 160 | 36 | 12 | 2.8 | 2.4 (1.2,4.8) |
| Females | 103 | 20 | 10 | 1.2 | 1.3 (0.6,3.0) |
| *Age* | | | | | |
| ⩽ 4 years | 64 | 1 | 1 | 3.0 | 3.2 (0.2,55.1) |
| – 14 | 181 | 48 | 21 | 1.8 | 1.6 (0.9,3.0) |
| > 15 | 18 | 7 | 0 | 2.2 | 3.5 (0.8,15.4) |
| *Tumour type* | | | | | |
| Leukaemia | 155 | 37 | 14 | 1.9 | 1.7 (0.9,3.3) |
| Wilms' tumour | 23 | 3 | 2 | 0.6 | 0.4 (0.0,3.4)[d] |
| Sarcomas | 31 | 8 | 4 | 3.0 | 2.7 (0.5,14.3) |
| Other tumours | 54 | 8 | 2 | 2.7 | 3.7 (0.8,17.0) |
| *Chemotherapy status* | | | | | |
| On maintenance | 101 | 14 | 10 | 1.9 | 1.3 (0.5,3.3) |
| Completed | 162 | 42 | 12 | 1.9 | 2.1 (1.1,4.0) |
| *Duration of treatment* | | | | | |
| ⩽ 1 month | 23 | 3 | 1 | 2.5 | 2.9 (0.5,17.9)[d] |
| 2–12 months | 96 | 17 | 6 | 3.2 | 2.6 (0.8,8.1) |
| > 12 months | 144 | 36 | 15 | 1.5 | 1.4 (0.8,2.6) |
| *Duration of remission* | | | | | |
| ⩽ 1 year | 40 | 8 | 4 | – | – |
| – 4 | 74 | 21 | 6 | 2.4 | 2.6 (1.0,7.0) |
| > 4 | 47 | 12 | 3 | 1.5 | 2.2 (0.8,6.4) |

[a]Adjusted for skin colour, propensity to freckle, history of sunburn, and time outdoors on weekends. [b]95% confidence limits. [c]Reference category for each variable is the subgroup with no acral naevi. [d]Adjusted for all variables in (a) except for skin colour due to limited number of pairs in analysis.. [e]No discordant pairs for analysis.

same sex and age. There was no apparent association between chemotherapy and the density of naevi overall in Queensland, however the acral subsite showed a significantly higher naevus density among childhood cancer survivors than controls. This had been hypothesised a priori, based on previous findings of a significant increase in acral naevi among children who had been treated for cancer in England (Hughes *et al.*, 1989). In addition, childhood cancer patients in Queensland who had received at least 4 weeks' chemotherapy had significantly more atypical naevi compared to matched controls.

While previous findings have been suggestive of a general association between chemotherapy and naevi, there is no firm evidence. Two case-control studies which found a positive association were based on relatively small numbers (Hughes *et al.*, 1989; de Wit *et al.*, 1990), while in a follow-up study which reported increased numbers of naevi in children 3 years after commencement of maintenance chemotherapy, there were potential problems with the comparison subjects who were ascertained some years earlier and whose naevi were counted using different criteria, in a cross-sectional study (Baird *et al.*, 1992). Furthermore, all these studies (Hughes *et al.*, 1989; de Wit *et al.*, 1990; Baird *et al.*, 1992) differed from the present study in that they were conducted in populations with low background levels of sun exposure. In Queensland children, a general association could indeed exist between chemotherapy and naevi, but it may be overshadowed by the effect of solar ultraviolet radiation. The very high overall prevalence of naevi in Queensland children compared to English children, for example, has been noted previously (Green *et al.*, 1988), and presumably reflects the large difference in ambient sun exposure in the two locations. However, regardless of location, the palms and soles are non-sunexposed sites, and it is reasonable to propose that chemotherapy may play a role in the development of acral naevi for which, like acral melanoma, causal factors are unknown. The apparent modifications of the increase in acral naevi in oncology patients by sex, by type of malignancy treated, and by duration of treatment in the present study are not easily explained and may be due to chance variation in these small subgroups. The additional association with atypical naevi suggests that abnormal melanocytic proliferation beyond the usual degree of proliferation associated with common naevi after sun exposure, occurs after chemotherapy. Mediation of the effect through the immune system has been suggested by Hughes *et al.* (1989), and it may be relevant that acral hyperpigmented macules are reported to occur in AIDS patients (Gallais *et al.*, 1992). An increased number of naevi found in 35 children with renal allografts compared with age- and sex-matched controls in a recent study in London (Smith *et al.*, 1991) offers further support for an immunosuppressive mechanism. Observation bias is unlikely to explain the study results because the research nurses, while not blinded to chemotherapy status, were unaware of any subtype hypotheses.

It has been suggested that survivors of childhood cancer should be especially counselled about the hazards of excessive sun exposure in relation to risk of melanoma (Hughes *et al.*, 1989; Baird *et al.*, 1992), and the occurrence of a melanoma in one of our study subjects would support such precaution. The development of this melanoma at an unusually young age and the increased occurrence of atypical naevi also support the need to carefully examine pigmented skin lesions as part of routine long-term surveillance of these children, although our data did not show an association between chemotherapy and naevi overall. The implications of a specific increase in acral naevi are less clear. It may be that these children are ultimately at risk of melanomas arising in idiopathic acral naevi. The relationship between acral-lentiginous melanoma and acral naevi has been studied in Ugandan Africans, but no direct association was observed (Lewis, 1968). However in view of the consistency in two analytic studies of an apparent excess of acral naevi in childhood cancer survivors, it may be appropriate to alert these patients to the significance in later life of any changes in plantar moles, since acral-lentiginous melanomas often have a poor prognosis due to late diagnosis.

This study was supported by the Queensland Cancer Fund. We are grateful to all patients and their parents who kindly participated in the study and to the hospital staff for their assistance.

### References

BAIRD, E.A., MCHENRY, P.M. & MACKIE, R.M. (1992). Effect of maintenance chemotherapy in childhood on numbers of melanocytic naevi. *Br. Med. J.*, **305**, 799-801.

BARKER, J.N.W.N. & MACDONALD, M. (1988). Eruptive dysplastic naevi following renal transplantation. *Clin. Exp. Dermatol.*, **13**, 123-125.

BRESLOW, N. & DAY, N.E. (1980). *Statistical Methods in Cancer Research* I. The analysis of case-control studies. I.A.R.C. Sci. Pub: Lyon.

CONOVER, J.W. (1980). *Practical Nonparametric Statistics*. John Wiley and Sons: New York.

DE WIT, P.E.J., DE VAAN, G.A.M., DE BOO, TH.M., LEMMENS, W.A.J.G. & RAMPEN, F.H.J. (1990). Prevlance of naevocytic naevi after chemotherapy for childhood cancer. *Med. Pediatr. Oncol.*, **18**, 336-338.

GALLAGHER, R., MCLEAN, D.I., YANG, C.P., COLDMAN, A.J., SILVER, H.K.B. & SPINELLI, J.J. (1990). Suntan, sunburn, and pigmentation factors and the frequency of acquired melanocytic nevi in children. *Arch. Dermatol.*, **126**, 770-776.

GALLAIS, V., LACOUR, J.P., PERRIN, C., GHANEM, G., BODOKH, I. & ORTONNE, J.P. (1992). Acral hyperpigmented macules and longitudinal melanonychia in AIDS patients. *Br. J. Dermatol.*, **126**, 387-391.

GREEN, A. & SWERDLOW, A.J. (1989). Epidemiology of melanocytic naevi. *Epidemiol. Reviews*, **11**, 204-221.

GREEN, A., SISKIND, V., HANSEN, M., HANSON, L. & LEECH, P. (1989). Melanocytic naevi in Queensland children. *J. Am. Acad. Dermatol.*, **20**, 1054-1060.

GREEN, A., SORAHAN, T., POPE, D., SISKIND, V., HANSEN, M., HANSON, L. LEECH, P., BALL, P.M. & GRIMLEY, R.P. (1988). Moles in Australian and British schoolchildren. *Lancet*, **2**, 1497.

HEYNE, K., HOF, M. & HANSEN, H.G. (1984). Pigmented naevi after therapy of leukaemia (ALL) in a monozygotic twin. *Eur. J. Pediatr.*, **142**, 70.

HUGHES, B.R. & BAILEY, C.C. (1989). Excess benign melanocytic naevi. *Br. Med. J.*, **299**, 854-855.

HUGHES, B.R., CUNLIFFE, W.J. & BAILEY, C.C. (1989). The development of excess numbers of benign melanocytic naevi in children after chemotherapy for malignancy. *Br. Med. J.*, **299**, 88-91.

IPPEN, H. & PRINDULL, G. (1984). Pigmented naevi after mercaptopurine. *Br. Med. J.*, **289**, 734.

LEWIS, M.G. (1968). The incidence and distribution of pigmented naevi in Ugandan Africans. *Br. J. Derm.*, **80**, 362-366.

MACKIE, R., ENGLISH, J., AITCHISON, T.C., FITZSIMONS, P.C. & WILSON, P.D. (1985). The number and distribution of benign pigmented moles (melanocytic naevi) in a healthy British population. *Br. J. Derm.*, **113**, 167-174.

MACKIE, R., HUNTER, J.A.A., AITCHISON, T.C., HOLE, D., MCLAREN, K., RANKIN, R., BLESSING, K., EVANS, A.T., HUTCHEON, A.W., JONES, D.H., SOUTAR, D.S., WATSON, A.C.H., CORNBLEET, M.A. & SMYTH, J.F. (1992). Cutaneous melanoma in Scotland, 1979-89. *Lancet*, **339**, 971-975.

MACLENNAN, R., GREEN, A., MARTIN, N. & MCLEOD, R. (1992). Increasing incidence of utaneous melanoma in Queensland. *J. Natl Cancer Inst.*, **84**, 1427-1432.

MEADOWS, A.T., KREJMAS, N.L. & BELASCO, J.B. (1980). The medical cost of cure: sequelae in survivors in childhood cancer. In *Status of the Curability of Childhood Cancers*, Van Eys, Sullivan (eds) pp. 263-276. Raven Press: New York.

MOSTELLER, R.D. (1987). Simplified calculation of body-surface area. *N. Engl. J. Med.*, **317**, 1098.

OCHS, J. & MULHERN, R.K. (1988). Late effects of antileukemic treatment. *Pediatr. Clin. North Am*,. **35**, 815-833.

QUEENSLAND CANCER REGISTRY. (1990). *Cancer in Queensland: Incidence and Mortality 1985*. Queensland Department of Health: Brisbane.

SMITH, C.H., MCGREGOR, J.M., BARKER, J.N., RIGDEN, S., MORRIS, R. & MACDONALD, D.M. (1991). Increase numbers of melanocytic naevi in children with renal allografts. *Br. J. Derm.*, **125**, 19 (S38).

STREILEIN, W.J. (1991). Immunogenetic factors in skin cancer. *N. Engl. J. Med.*, **325**, 884-887.